# Classification and Interpretability of Mild Cognitive Impairment Based on Resting-State Functional Magnetic Resonance and Ensemble Learning

**DOI:** 10.1155/2022/2535954

**Published:** 2022-08-19

**Authors:** Mengjie Hu, Yang Yu, Fangping He, Yujie Su, Kan Zhang, Xiaoyan Liu, Ping Liu, Ying Liu, Guoping Peng, Benyan Luo

**Affiliations:** ^1^Department of Neurology, First Affiliated Hospital, Zhejiang University School of Medicine, Hangzhou 310003, China; ^2^Department of General Practice, First Affiliated Hospital, Zhejiang University School of Medicine, Hangzhou 310003, China

## Abstract

The combination and integration of multimodal imaging and clinical markers have introduced numerous classifiers to improve diagnostic accuracy in detecting and predicting AD; however, many studies cannot ensure the homogeneity of data sets and consistency of results. In our study, the XGBoost algorithm was used to classify mild cognitive impairment (MCI) and normal control (NC) populations through five rs-fMRI analysis datasets. Shapley Additive exPlanations (SHAP) is used to analyze the interpretability of the model. The highest accuracy for diagnosing MCI was 65.14% (using the mPerAF dataset). The characteristics of the left insula, right middle frontal gyrus, and right cuneus correlated positively with the output value using DC datasets. The characteristics of left cerebellum 6, right inferior frontal gyrus, opercular part, and vermis 6 correlated positively with the output value using fALFF datasets. The characteristics of the right middle temporal gyrus, left middle temporal gyrus, left temporal pole, and middle temporal gyrus correlated positively with the output value using mPerAF datasets. The characteristics of the right middle temporal gyrus, left middle temporal gyrus, and left hippocampus correlated positively with the output value using PerAF datasets. The characteristics of left cerebellum 9, vermis 9, and right precentral gyrus, right amygdala, and left middle occipital gyrus correlated positively with the output value using Wavelet-ALFF datasets. We found that the XGBoost algorithm constructed from rs-fMRI data is effective for the diagnosis and classification of MCI. The accuracy rates obtained by different rs-fMRI data analysis methods are similar, but the important features are different and involve multiple brain regions, which suggests that MCI may have a negative impact on brain function.

## 1. Introduction

Mild cognitive impairment (MCI) is a heterogeneous syndrome that causes little or no impairment of daily living activities and thus does not meet the criteria for dementia [1, 2]. Among the aging population (60 years of age and above) in China, the prevalence of MCI is 14.71%; besides, females of older age or living in rural areas of western China have a higher prevalence of MCI [3]. The currently available diagnoses of MCI are based on subjective indicators, including observation, clinical history, and neuropsychological assessment; moreover, its reliable diagnosis is challenging [4]. Approximately 30% of MCI patients progress to AD [5]. Early diagnosis and intervention delay the transformation of MCI to AD and improve its prognosis [6].

Machine learning is extensively used in the clinical and early diagnosis of diseases. Dichotomous and tripartite diagnosis is the most basic application in Alzheimer's Disease (AD), i.e., diagnosis of AD and normal control (NC), as well as that of AD, MCI, and NC. Notably, classification diagnosis based on these two types is still in use today [7].

Several types of data commonly used in machine learning include structure magnetic resonance imaging (sMRI), positron emission tomography (PET), and resting-state functional magnetic resonance imaging (rs-fMRI). Rs-fMRI shows characteristic focal changes of AD, including reduced hippocampal volume and medial temporal lobe atrophy [8]; it excludes other diseases that may cause dementia, including cerebrovascular diseases and other structural diseases (such as brain tumors and normal pressure hydrocephalus). FDG-PET shows decreased metabolism in different areas of AD, including the hippocampus, medial parietal lobe, and lateral parietal cortex [9, 10]. Rs-fMRI is highly sensitive to AD and is used to analyze changes in brain networks in AD patients. Additionally, accumulating studies indicate that internal connection in the resting state provides a communication channel for task information [11].

Rs-fMRI is a noninvasive imaging method with a high spatial and temporal resolution, continually adopted in scientific research and clinical work. Rs-fMRI primarily reflects neuronal activity by observing the blood oxygen level–dependent (BOLD) signal changes. The spontaneous activity of neurons may trigger low-frequency fluctuation (LFF). Studies integrating neuron electrophysiology and rs-fMRI reveal that many cognitive and behavioral processes are related to LFF [12–14]. Biswal et al. [15] discovered a highly synchronous spontaneous LFF between motor cortices, and the LFF of a BOLD signal is closely related to neuronal spontaneous activity and is used to reflect changes in brain functional activities. Changes in the amount of LFF in different brain regions may be related to the interruption of automatic regulation of the cerebral microvascular system [16]. Numerous studies on LFF, including low-frequency fluctuation (ALFF) [17], fractional ALFF (fALFF) [13], percent amplitude of fluctuation (PerAF) [18], Wavelet-ALFF [19], have been documented.

At present, studies on machine learning- (ML-) based diagnosis studies with rs-fMRI have reached maturity (Table 1). Most of the studies focuses on the interpretability of models and the improvement of feature extraction methods and classification algorithm. The accuracy of some predictive models has reached more than 90%. Nevertheless, because of the small sample size, the credibility of these studies is at stake.

NC: normal controls; eMCI: early MCI; lMCI: late MCI; aMCI: amnestic MCI; MCI-C : MCI converter, MCI-NC : MCI nonconverter; SCD: subjective cognitive decline; VD: vascular dementia; MXD: “mixed VD-AD dementia”; CNN: convolutional neural network; SVM: support vector machine; LDA: linear discriminant analysis; RF: random forest; ANFIS: adaptive neurofuzzy inference system; ELM: extreme learning machine; DAG: directed acyclic graph; AE: autoencoder.

Herein, we established a database of rs-fMRI studies involving MCI and NC based on the local population; this increased the applicability of the findings. Besides, the combination and integration of multimodal imaging and clinical markers have elicited numerous classifiers that improve diagnostic accuracy in detecting and predicting AD or MCI. Although the accuracy obtained is significantly attractive, numerous studies cannot guarantee data homogeneity and consistent results [45]. We used the XGBoost algorithm to classify MCI and NC populations, and the results were explained.

## 2. Materials and Methods

### 2.1. Participants

Between January 2017 and December 2020, patients were recruited from the Memory Clinic of the First Affiliated Hospital, Zhejiang University School of Medicine. Eligible participants were aged 55 years or older, with primary school education or above. Peterson's criteria were used to select the MCI patients [46]. Individuals were excluded if they had evidence of other diseases potentially causing dementia other than AD; a history of stroke and focal signs of nervous system; other neurological diseases that potentially cause brain dysfunction (including schizophrenia, severe anxiety, depression, frontotemporal dementia, Huntington's disease, brain tumors, Parkinson's disease, metabolic encephalopathy, encephalitis, multiple sclerosis, epilepsy, and brain trauma); other systemic diseases that potentially cause cognitive impairment including hypothyroidism, folic acid and vitamin B_12_ deficiency, specific infections (e.g., syphilis and HIV), and alcohol and drug abuse; severe liver, kidney and lung insufficiency; severe anemia, gastrointestinal disease and arrhythmia, and myocardial infarction within 6 months; contraindications including metal implantation *in vivo*; aphasia, consciousness disorders, and other diseases that potentially hinder the completion of cognitive examination; did not sign informed consent. This study was authorized and approved by the Ethics Committee of First Affiliated Hospital, Zhejiang University School of Medicine, and conducted based on the principles of the Helsinki Declaration. After obtaining informed consent, participants were subjected to initial tests, including clinical evaluation, neuropsychological tests, laboratory examination, and MRI scanning.

### 2.2. Data Acquisition

All rs-fMRI data were collected from the Second Affiliated Hospital of Hangzhou Normal University from a Discovery MR750 3.0 T scanner of General Electric Company. Rs-fMRI scans were acquired based on the following parameters: 43 slices, TR = 2000 ms, TE = 30 ms, FA = 90, FOV = 64 mm × 64 mm, matrix = 200 × 200, scanning time = 8 min.

### 2.3. Rs-fMRI Data Preprocessing

Data preprocessing was performed using the RESTplus V1.2 tool [47] (http://www.restfmri.net/forum/RESTplusV1.2) in the SPM12 (Statistical Parametric Mapping 12) (http://www.fil.ion.ucl.ac.uk/spm). The rs-fMRI data preprocessing steps included the following: (1) removing volumes, i.e., the first ten volumes of each subject were removed to ensure a steady condition; (2) slice timing, i.e., data scanning was performed in intervals, with odd-numbered layers having priority; (3) realignment, i.e., subjects with a maximum translation of more than 3.0 mm or maximum rotation of more than 3.0° were excluded; (4) normalization, i.e., the rs-fMRI scans were registered to correspond sMRI and split using the Diffeomorphic Anatomical Registration Through Exponentiated Lie Algebra (DARTEL) and new segment, which were spatially normalized to the Montreal Neurological Institute (MNI) space; (5) detrend, i.e., the offset generated during data acquisition may have an impact on the later calculation process. Detrend eliminates such an impact when the data is acquired; (6) Nuisance Covariates Regression, i.e., factors affecting the results were removed, including Friston24 rotation parameter, white matter, cerebrospinal fluid, and global mean signal [48, 49].

### 2.4. Rs-fMRI Features Extraction

Further, features extraction of rs-fMRI was performed using RESTplus V1.2; consequently, 116 features based on Anatomical Automatic Labeling (AAL) were extracted in each type of calculation method. The features included fractional amplitude of low-frequency fluctuations (fALFF). The preprocessed data results were registered into the MNI space; then, each voxel was resampled using a sampling template of 3 mm × 3 mm × 3 mm. In RESTplus, BOLD was transformed from a time domain to a frequency domain by the fast Fourier transform formula (FFT), and the power spectrum of the BOLD signal in the frequency domain was obtained. The power spectrum obtained was calculated via square root, and the result obtained by calculating the mean value of the effective frequency band divided by a mean value of the amplitude of the whole frequency band was fALFF. Subsequently, the spatial fALFF maps were divided by the mean value of the whole brain (mfALFF). This study calculated fALFF in three frequency bands, i.e., Norm-1 (0.01–0.08 Hz), Slow-4 (0.027–0.073 Hz), and Slow-5 (0.01–0.027 Hz) frequency bands. A Gaussian smoothing kernel of 4 mm full width-half maximum (FWHM) was selected to improve the signal-to-noise ratio of the data.

#### 2.4.1. PerAF and mPerAF

Based on the formula, PerAF was calculated by subtracting the BOLD signal intensity of each voxel from the mean-time series value of the voxel and then dividing by the mean-time series value. Then the sum of absolute values of each voxel in the time series was divided by the number of time points to obtain the percentage of the fluctuation relative to the mean BOLD signal intensity, namely, the PerAF value of each time series. Unlike ALFF, fALFF, and PerAF, the results are directly used for comparison or can be compared after averaging (mPerAF). This study calculated mPerAF and PerAF in three frequency bands, i.e., Norm-1, Slow-4, and Slow-5 frequency bands. A Gaussian smoothing kernel of 4 mm FWHM was selected to improve the signal-to-noise ratio of data.

#### 2.4.2. Wavelet-ALFF

The continuous wavelet transform was performed on data, and the convolution of scaling and translation form of the mother wavelet function was calculated. Then, the coefficients of each frequency point at all-time points were added for calculation, then the average coefficient of a given frequency band was obtained. This study calculated Wavelet-ALFF in three frequency bands, i.e., Norm-1, Slow-4, and Slow-5. A Gaussian smoothing kernel of 4 mm FWHM was selected to improve the signal-to-noise ratio of data.

#### 2.4.3. Degree Centrality (DC)

Other nodes with significant functional connection (*r* > 0.25) with each node in each brain functional connection group were calculated to obtain the sum DC value of the significant correlation weight of each node, then divided by the average DC value of the whole brain to obtain the standardized DC value. This study calculated DC in three frequency bands, including Norm-1 (0.01–0.08 Hz), Slow-4 (0.027–0.073 Hz), and Slow-5 (0.01–0.027 Hz) frequency bands. A Gaussian smoothing core of 4 mm full width-half maximum (FWHM) was selected to improve the signal-to-noise ratio of data.

### 2.5. Statistical Analysis

SPSS 23.0 software was used for statistical analysis in the demographic statistics part of this study. Categorical variables, including gender, were marked with the number of each group for direct description. Age, education level, scale score, and other continuous variables were described as mean ± standard deviation (SD) for the MCI group and NC group. An independent sample *t-*test or Chi-square test was used for comparison between the two groups.

### 2.6. Extreme Gradient Boosting (XGBoost) Classifier

XGBoost is a type of composite tree model comprising a series of regression and classification trees. As an open source package, XGBoost is widely recognized in many machine learning and data mining challenges, for example, 17 out of 29 challenge solutions posted on the Kaggle blog in 2015 used XGBoost, and the top 10 winning teams in the 2015 KDD Cup used XGBoost [50]. PyCaret 2.1 in Jupyter Notebook was used to train and validate the XGBoost classifier.

## 3. Results

### 3.1. Demographics Differences among NC and MCI Groups

The demographic characteristics of study participants are shown in Table 2. The MMSE score (NC: 28.53 ± 1.248, MCI: 25.47 ± 2.506, *p* < 0.001) and MoCA score (NC: 26.23 ± 1.820, MCI: 19.60 ± 2.768, *p* < 0.001) were significantly different among groups, while no significant differences were noted in age, gender ratio, and education level.

Independent-samples *t*-test was used to examine the differences in the characteristics of NC and MCI groups and categorical data were compared using X^2^ tests. ^*∗*^Statistically significant differences (*p* < 0.05).

### 3.2. Classification Performance

The XGBoost classifier was trained and validated using 10-fold cross-validation to estimate out-of-sample performance. AUC, recall rate, precision, F1-score, Kappa value, and accuracy were reported.  Table 3 shows binary classification performances of the XGBoost classifier in feature datasets. The results revealed that lower levels of accuracy were achieved in all comparisons. The highest accuracy (65.14%) was observed in the mPerAF datasets. Highest AUC (0.6608), recall rate (53.33%), and F1-score (0.5285) were obtained in the fALFF datasets. The highest precision (60.00%) was obtained in the DC datasets. The highest Kappa value (0.2191) was obtained in Wavelet-ALFF datasets.

**Figure 1:**
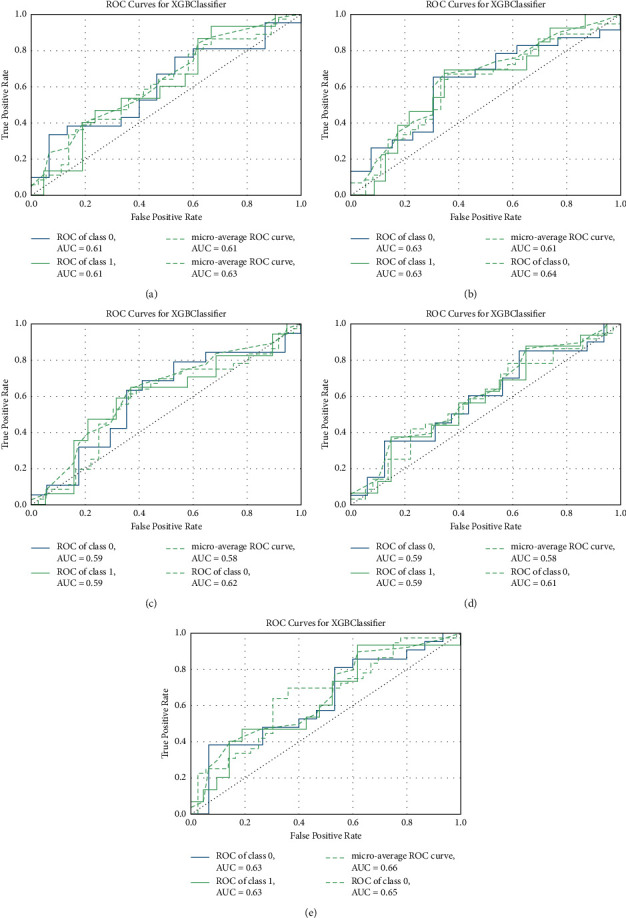
ROC curves of XGBoost classifier trained on 90% of datasets and tested on the remaining 10% of datasets. ROC curves of XGBoost classifier on (a) DC datasets, (b) fALFF datasets, (c) mPerAF datasets, (d) PerAF datasets, and (e) Wavelet-ALFF datasets.

The receiver operating characteristic (ROC) curves of the XGBoost classifier trained on 90% of datasets and tested on the remaining 10% of datasets are shown in Figure 1. The AUC of the micro-average ROC curve and macro-average ROC for prediction using DC datasets were 0.61 and 0.63 (Figure 1(a)). The AUC of the micro-average ROC curve and macro-average ROC for prediction using fALFF datasets were 0.61 and 0.64 (Figure 1(b)). The AUC of the micro-average ROC curve and macro-average ROC for prediction using mPerAF datasets were 0.58 and 0.62 (Figure 1(c)). The AUC of the micro-average ROC curve and macro-average ROC for prediction using PerAF datasets were 0.58 and 0.61 (Figure 1(d)). The AUC of the micro-average ROC curve and macro-average ROC for prediction using Wavelet-ALFF datasets were 0.66 and 0.65 (Figure 1(e)).

### 3.3. Model Interpretation: Shapley Additive exPlanations (SHAP)

**Figure 2:**
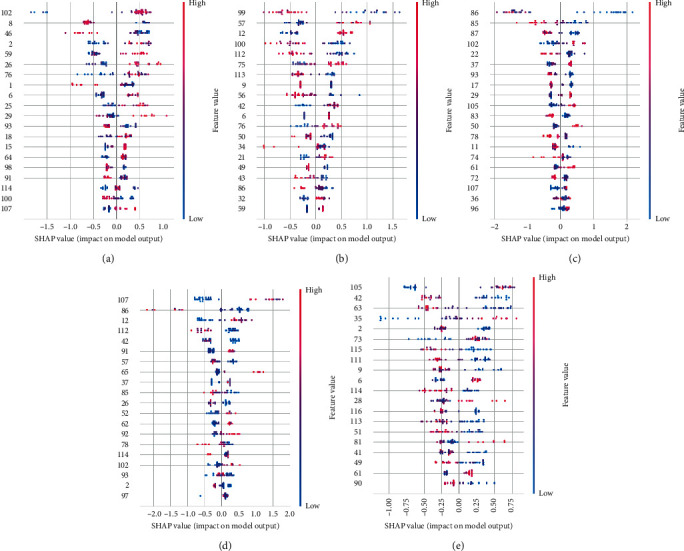
The XGBoost classifier using (a) DC datasets, (b) fALFF datasets, (c) mPerAF datasets, (d) PerAF datasets, and (e) Wavelet-ALFF datasets based on the SHAP algorithm.

Anatomical Automatic Labeling (AAL) is provided by Montreal Neurological Institute (MNI), with a total of 116 regions. A total of 90 regions belong to the brain, while the remaining 26 regions belong to the cerebellum. Each region has the MRIcro number from 1 to 116. Based on the SHAP algorithm, the feature ranking interpretation of the XGBoost classifier shows the top 20 great characteristics in predicting outcomes with different datasets (Figure 2). Generally, the characteristics of the superior parietal gyrus (59), parahippocampal gyrus (40), right cerebellum 7b (102), inferior temporal gyrus (90), and right superior frontal gyrus, orbital part (6) positively correlated with the outcomes using DC datasets. The characteristics of left cerebellum 6 (99), left postcentral gyrus (57), right inferior frontal gyrus, opercular part (12), right cerebellum 6 (100), and vermis 6 (112) positively correlated with outcomes using fALFF datasets. The characteristics of the right middle temporal gyrus (86), left middle temporal gyrus (85), left temporal pole: middle temporal gyrus (87), right cerebellum 7b (102), and right olfactory cortex (22) positively correlated with outcomes using mPerAF datasets. The features of the left cerebellum 10 (107), right middle temporal gyrus (86), right inferior frontal gyrus, opercular part (12), vermis 6 (112), and right amygdala (42) positively correlated with outcomes using PerAF datasets. The characteristics of left cerebellum 9 (105), right amygdala (42), left supramarginal gyrus (63), left posterior cingulate gyrus (35), and right precentral gyrus (2) positively correlated with outcomes using Wavelet-ALFF datasets.

**Figure 3:**
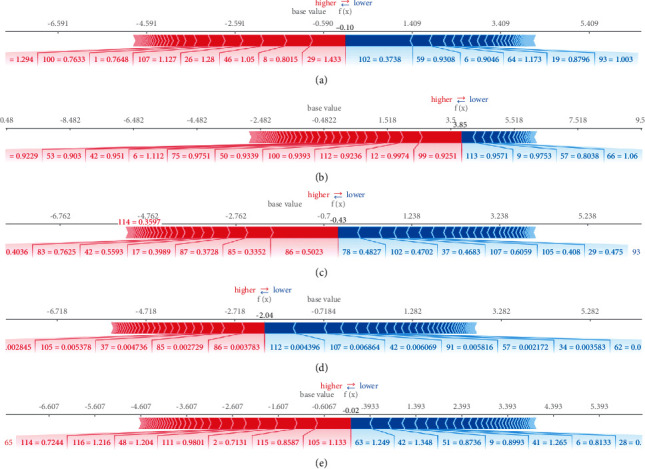
Force plot based on the SHAP algorithm. The XGBoost classifier using (a) DC datasets, (b) fALFF datasets, (c) mPerAF datasets, (d) PerAF datasets, and (e) Wavelet-ALFF datasets.

SHAP force plot (Figure 3) shows the interpretability of a single model prediction used for error analysis to identify an interpretation for a particular instance prediction. The output value of the XGBoost classifier using DC datasets was −0.10, and the characteristics of the left insula (29), right middle frontal gyrus (8), and right cuneus (46) positively correlated with the output value, whereas the characteristics of the right cerebellum 7b (102), left superior parietal gyrus (59), right superior frontal gyrus, and orbital part (6) negatively correlated with the output value. The output value of the XGBoost classifier using fALFF datasets was 3.85 and features of left cerebellum 6 (99), right inferior frontal gyrus, opercular part (12), and vermis 6 (112) positively correlated with the output value, whereas the characteristics of vermis 7 (113), left middle frontal gyrus, orbital part (9), and left postcentral gyrus (57) negatively correlated with the output value. The output value of the XGBoost classifier using mPerAF datasets was −0.43; the characteristics of the right middle temporal gyrus (86), left middle temporal gyrus (85), left temporal pole, and middle temporal gyrus (87) positively correlated with the output value, while the characteristics of right thalamus (78), right cerebellum 7b (102), and left hippocampus (37) negatively correlated with the output value. The output value of the XGBoost classifier using PerAF datasets was −2.04; the characteristics of the right middle temporal gyrus (86), left middle temporal gyrus (85), and left hippocampus (37) positively correlated with the output value, whereas the characteristics of vermis 6 (112), left cerebellum 10 (107), and right amygdala (42) negatively correlated with the output value. The output value of the XGBoost classifier using Wavelet-ALFF datasets was −0.02 and the characteristics of left cerebellum 9 (105), vermis 9 (115), and right precentral gyrus (2) correlated positively with the output value, whereas the characteristics of the left supramarginal gyrus (63), right amygdala (42), and left middle occipital gyrus (51) negatively correlated with the output value.

## 4. Discussion

A total of 15 machine learning models were used in each of the five datasets (see Tables S1–S5 in the Supplementary Materials for classification performance on the five datasets), and eventually, XGBoost algorithm was selected for the classification diagnosis of MCI and NC based on the overall performance and interpretability of the model. Besides, we used 116 features from rs-fMRI analysis in model classification diagnosis. Based on the analysis of model performance, it was difficult to classify MCI and NC using rs-fMRI features alone, and the highest accuracy was only 65.14% (using the mPerAF dataset).

In contrast with the classification of AD and NC, the classification of MCI and NC is more difficult but appears meaningful because although AD cannot be cured, intervention in MCI patients effectively delays recognition and decreases cognitive capacity [51]. In previous studies, unlike the classification diagnosis of AD and NC, the diagnostic accuracy of MCI and NC is lower. For instance, Lama and Kwon [52] used functional magnetic resonance image features based on graph theory for classification. In the classification diagnosis of MCI and NC, although the accuracy rate of 97.80% is obtained using Lasso regression, only 80%–86% accuracy rate is obtained when other algorithms, including support vector machine based on feature elimination, adaptive structure learning, feature learning based on pairwise correlation are used. Bergeron et al. [53] used the MemTrax test combined with the MoCA score to make model predictions and obtained a prediction accuracy of approximately 90%. Unlike the accuracy rates reported in other studies, our accuracy rate is lower; this is potentially attributed to the overfitting of the XGBoost algorithm model because of the small sample size.

Based on our XGBoost algorithm model, the results are quite different when applied to different datasets. The important features correspond to the following AAL regions, i.e., superior parietal gyrus (59), parahippocampal gyrus (40), right cerebellum 7b (102), left cerebellum 6 (99), left postcentral gyrus (57), right inferior frontal gyrus, opercular part (12), right middle temporal gyrus (86), left middle temporal gyrus (85), left temporal pole: middle temporal gyrus (87), left cerebellum 10 (107), vermis 6 (112), left cerebellum 9 (105), right amygdala (42), and left supramarginal gyrus (63). We counted the top 20 features in five SHAP algorithm graphs. Among the 116 features, 66 features appeared in the graph, most of which only appeared in SHAP of a certain dataset, among which right cerebellum 7b (102), right superior frontal gyrus, orbital part (6), right middle temporal gyrus (86), right amygdala (42), and vermis 6 (112) appeared with high frequency (≥3). This suggests that the effect of disease state on brain function is extensive, and comprehensive analysis combined with multiple indicators may be beneficial to further analyze the mechanism of cognitive impairment.

Previous studies on MCI indicate that patients with MCI have posterior cingulate gyrus, cuneus, superior marginal gyrus, hippocampus (belonging to the default network), insula (belonging to the prominence network), the lingual gyrus, middle occipital gyrus, and inferior temporal gyrus (belonging to the visual network), which are different from NC [54, 55]. Lenka [56] et al. applied psychophysiological interaction (PPI) analysis to detect specific alterations in PCC connectivity associated with visual processing while controlling brain atrophy. This approach separated the MCI from NC with 77% sensitivity and 89% specificity. Suh et al. [57] developed a 2-step algorithm using a convolutional neural network to perform brain parcellation followed by 3 classifier techniques, including XGBoost for disease prediction. Compared with SVM and logistic regression, XGBoost had a sensitivity of 68% and a specificity of 70% in terms of differentiating AD from the MCI group. In terms of MCI from the NC group, XGBoost had a sensitivity of 79% and a specificity of 80%. Shmulev et al. [58] used brain MRI and clinical data to predict MCI conversion to the AD. The resulting accuracy by the XGBoost algorithm is 0.76 ± 0.01, and the AUC is 0.86 ± 0.01. Jo et al. [59] proposed a novel three-step approach (SWAT-CNN) for the identification of genetic variants using deep learning to identify phenotype-related single nucleotide polymorphisms (SNPs) that can be applied to develop accurate disease classification models, and the AUC of this model is 0.82. A machine learning framework proposed in this paper for MCI detection achieved an accuracy of 65.14% when using the mPerAF dataset. These findings provide novel insights into the understanding of pathological changes in the brain functional network organization of MCI and show the potential of the PerAF analysis-related features for MCI detection.

In the present study, we found abnormal cerebellar activation in all five datasets. In the past, the cerebellum was primarily associated with voluntary movement and postural balance. However, clinical and anatomical work suggests that the cerebellum may also play a role in cognition [60]. A significant number of fMRI research reports further provide supporting evidence that the cerebellum is activated to varying degrees in cognitive tasks (including language, working memory, and spatial processing) [61, 62]. Studies have proposed a connection system between the cerebellum and thalamus; the cerebellum is connected with the thalamus through the brain stem and participates in related functions of the frontal lobe cognitive circuit [63].

This work has compelling shortcomings. First, as a single-center study, only 117 subjects were included; this is a relatively small sample size; thus, a larger sample is required in subsequent studies to further verify the stability of the results. Secondly, the study lacks long-term follow-up, which makes it impossible to track the conversion of MCI. In contrast with the classification diagnosis of MCI and NC, predicting the conversion of MCI in practical application is necessary. Lastly, this study lacks PET-CT/MR radionuclide (such as AV-45) labeling results of subjects, thus limiting its credibility and conviction. Therefore, it is necessary to cooperate with multiple hospitals to perform multicenter researches. Besides, reliable pathological diagnosis, including the improvement of A*β* protein examination of cerebrospinal fluid or AV-45 PET, improves the value of the results in clinical application.

## 5. Conclusion

In conclusion, our findings demonstrate that the XGBoost algorithm constructed from rs-fMRI data is effective in classifying and diagnosing MCI. Using mPerAF dataset, we obtained the highest accuracy for diagnosing MCI. This suggests that the outcomes of rs-fMRI analysis may be useful as imaging markers for MCI diagnosis. The accuracy rates obtained by different rs-fMRI data analysis methods are similar, but the important features are different and involve multiple brain regions, which suggests that MCI may have a negative impact on brain function.

## Figures and Tables

**Table 1:** Partial ML-based studies with rs-fMRI.

Year	ML method	Subjects	Performance
2022 [20]	CNN	NC: 167, eMCI: 102, lMCI: 129, AD: 114	Average accuracy 89%
2022 [21]	AdaBoost	eMCI: 34, lMCI: 32	Accuracy 70%
2022 [22]	SVM	NC: 20, AD: 27	Accuracy (fMRI) 78.72%
			Accuracy (sMRI + rs-fMRI) 91.49%
2022 [23]	SVM	NC: 41, aMCI: 30, AD: 36	Accuracy (NC vs. aMCI) 68%
			Accuracy (NC vs. AD) 71%
2022 [24]	LDA	NC: 30, AD: 28	Accuracy 76.7%
2021 [25]	SVM	MCI-C: 14, MCI-NC: 41	Accuracy (fMRI) 83.5%
			Accuracy (sMRI + rs-fMRI) 83.5%
2021 [26]	SVM	MCI-C: 30, MCI-NC: 55, AD: 19	Accuracy (MCI-C vs. MCI-NC) 84.71%
			Accuracy (MCI-C vs. AD) 89.80%
2020 [27]	SVM	NC: 51, MCI: 66	Accuracy 85.5%
2020 [28]	SVM	NC: 20, SCD: 22	Accuracy 83.3%
2020 [29]	RF	NC: 83, MCI: 82	Accuracy 91.4%
2020 [30]	SVM	NC: 136, SMC: 46, eMCI: 83, MCI: 37, lMCI: 46, AD: 35	AUC (AD vs. NC) 0.87
2020 [31]	ANFIS	AD: 33, VD: 27, MXD: 15	Average accuracy 77.33%
2020 [32]	SVM	eMCI: 77, lMCI: 64	Accuracy 87.94%
2020 [33]	SVM	NC: 60, MCI: 39	AUC 0.9728
2019 [34]	ResNet-18	NC: 25, SMC: 25, eMCI: 25, lMCI: 25, MCI: 13, AD: 25	Average accuracy 97.88%
2019 [11]	SVM	NC: 49, MCI-NC: 69, MCI-C: 25, AD: 34	Accuracy (AD vs. MCI-C vs. MCI-NC) 67.6%
			Accuracy (NC vs. MCI-C vs. MCI-NC) 66%
			Accuracy (AD vs. NC vs. MCI-C vs. MCI-NC) 56.1%
2019 [35]	SVM	NC: 45, AD: 45	Accuracy 81.11%
2019 [36]	CNN	NC: 172, eMCI: 179	Accuracy 73.85%
2020 [37]	CNN	NC: 198, AD: 133	Accuracy 85.27%
2019 [38]	SVM	NC: 45, SCD: 39, aMCI: 45, AD: 38	Accuracy (AD vs. NC) 98.58%
			Accuracy (aMCI vs. NC) 97.76%
			Accuracy (SCD vs. NC) 80.24%
2019 [39]	SVM	NC: 24, eMCI: 24, lMCI: 24, AD: 24	Accuracy (eMCI vs. NC) 93.8%
			Accuracy (lMCI vs. NC) 95.8%
			Accuracy (AD vs. NC) 95.8%
			Accuracy (eMCI vs. lMCI) 87.5%
			Accuracy (lMCI vs. AD) 91.7%
2019 [40]	ELM	NC: 31 + 152, MCI: 31 + 132, AD: 33 + 81	In ANDI-2 cohort: Accuracy (AD vs. NC) 94.07%
			Accuracy (MCI vs. NC) 87.54%
			In the in-house cohort: Accuracy (AD vs. NC) 95.5%
			Accuracy (MCI vs. NC) 86.52%
2018 [41]	DAG network	NC: 34, AD: 34	Accuracy 95.59%
2018 [42]	SVM	MCI-C: 18, MCI-NC: 62	Accuracy 97%
2019 [43]	AE	NC: 79, MCI: 91	Accuracy 86.47%
2018 [44]	SVM	NC: 35, AD: 25	Accuracy 94.44%

**Table 2:** Demographics of datasets.

	NC (*n* = 47)	MCI (*n* = 70)	*p* value
Age (years)	64.91 ± 9.029	66.47 ± 8.007	0.72
Gender (M/F)	17/30	36/34	0.131
Education (years)	12.53 ± 2.896	9.10 ± 3.046	0.582
MMSE	28.53 ± 1.248	25.47 ± 2.506	<0.001^*∗*^
MoCA	26.23 ± 1.820	19.60 ± 2.768	<0.001^*∗*^

**Table 3:** Classification performance of XGBoost classifier.

	Accuracy (%)	AUC	Recall (%)	Precision (%)	F1-score	Kappa
DC	62.78	0.6558	40.00	**60.00**	0.4538	0.1803
fALFF	61.94	**0.6608**	**53.33**	58.33	**0.5285**	0.2086
mPerAF	**65.14**	0.6333	40.00	54.00	0.4243	0.2077
PerAF	57.78	0.5867	34.17	46.67	0.3802	0.0742
Wavelet-ALFF	63.33	0.6142	48.33	55.83	0.4833	**0.2191**

## Data Availability

The rs-fMRI data used to support the findings of this study are restricted by the Ethics Committee of First Affiliated Hospital, Zhejiang University School of Medicine, in order to protect patient privacy. Data are available from the corresponding author for researchers who meet the criteria for access to confidential data.

## Supplementary Materials

Supplementary Materials Tables S1–S5 in the Supplementary Material show comparison of the performance of 15 machine learning models using the DC/fALFF/mPerAF/PerAF/Wavelet-ALFF dataset.
