# Supplementary material for: Classification and Interpretability of Mild Cognitive Impairment Based on Resting-State Functional Magnetic Resonance and Ensemble Learning
Source: Comput Intell Neurosci. 2022 Aug 19;2022:2535954. doi: 10.1155/2022/2535954 (PMC9417789; doi:10.1155/2022/2535954)
Supplement: Supplementary Materials — Tables S1–S5 in the Supplementary Material show comparison of the performance of 15 machine learning models using the DC/fALFF/mPerAF/PerAF/Wavelet-ALFF dataset. [file 2535954.f1.docx]

Table S1 Classification Performance using DC dataset

| **Model** | **Accuracy** | **AUC** | **Recall** | **Prec.** | **F1** | **Kappa** | **MCC** |
| --- | --- | --- | --- | --- | --- | --- | --- |
| **Naive Bayes** | 0.6681 | 0.6150 | 0.6000 | 0.5917 | 0.5810 | 0.3153 | 0.3290 |
| **Ada Boost Classifier** | 0.6306 | 0.6425 | 0.5000 | 0.5417 | 0.5064 | 0.2169 | 0.2245 |
| **Gradient Boosting Classifier** | 0.6278 | 0.6300 | 0.4667 | 0.5700 | 0.4705 | 0.2123 | 0.2419 |
| **Extreme Gradient Boosting** | 0.6278 | 0.6558 | 0.4000 | 0.6000 | 0.4538 | 0.1803 | 0.2083 |
| **Ridge Classifier** | 0.6181 | 0.0000 | 0.4417 | 0.5867 | 0.4917 | 0.1979 | 0.2077 |
| **Logistic Regression** | 0.6056 | 0.6425 | 0.3167 | 0.6200 | 0.3983 | 0.1374 | 0.1692 |
| **Light Gradient Boosting Machine** | 0.6056 | 0.6354 | 0.3750 | 0.5167 | 0.4110 | 0.1359 | 0.1513 |
| **Random Forest Classifier** | 0.5917 | 0.5262 | 0.2167 | 0.5333 | 0.2967 | 0.0670 | 0.1049 |
| **CatBoost Classifier** | 0.5792 | 0.6321 | 0.2167 | 0.4500 | 0.2700 | 0.0419 | 0.0763 |
| **Extra Trees Classifier** | 0.5667 | 0.5525 | 0.1917 | 0.3833 | 0.2433 | 0.0107 | 0.0146 |
| **Decision Tree Classifier** | 0.5556 | 0.5558 | 0.5417 | 0.4375 | 0.4665 | 0.1024 | 0.1045 |
| **SVM - Linear Kernel** | 0.5556 | 0.0000 | 0.3333 | 0.2762 | 0.2800 | 0.0091 | 0.0226 |
| **Quadratic Discriminant Analysis** | 0.5319 | 0.5267 | 0.5083 | 0.4133 | 0.4528 | 0.0445 | 0.0491 |
| **K Neighbors Classifier** | 0.5069 | 0.4079 | 0.1583 | 0.2000 | 0.1733 | -0.116 | -0.140 |
| **Linear Discriminant Analysis** | 0.5069 | 0.4612 | 0.5083 | 0.4150 | 0.4464 | 0.0261 | 0.0233 |

Table S2 Classification Performance using fALFF dataset

| **Model** | **Accuracy** | **AUC** | **Recall** | **Prec.** | **F1** | **Kappa** | **MCC** |
| --- | --- | --- | --- | --- | --- | --- | --- |
| **Ridge Classifier** | 0.6819 | 0.0000 | 0.4750 | 0.6333 | 0.5174 | 0.3066 | 0.3236 |
| **CatBoost Classifier** | 0.6444 | 0.6550 | 0.5083 | 0.5233 | 0.4990 | 0.2446 | 0.2486 |
| **Naive Bayes** | 0.6306 | 0.6046 | 0.7667 | 0.5500 | 0.6267 | 0.2787 | 0.3154 |
| **Extra Trees Classifier** | 0.6194 | 0.6415 | 0.3833 | 0.4833 | 0.4210 | 0.1727 | 0.1727 |
| **Extreme Gradient Boosting** | 0.6194 | 0.6608 | 0.5333 | 0.5833 | 0.5285 | 0.2086 | 0.2303 |
| **Light Gradient Boosting Machine** | 0.6194 | 0.6567 | 0.4667 | 0.5400 | 0.4914 | 0.1931 | 0.1943 |
| **Random Forest Classifier** | 0.6083 | 0.6546 | 0.4167 | 0.5150 | 0.4340 | 0.1632 | 0.1770 |
| **K Neighbors Classifier** | 0.5944 | 0.6729 | 0.4583 | 0.5267 | 0.4843 | 0.1437 | 0.1502 |
| **Ada Boost Classifier** | 0.5819 | 0.5392 | 0.4750 | 0.4417 | 0.4356 | 0.1134 | 0.1173 |
| **Linear Discriminant Analysis** | 0.5819 | 0.6254 | 0.5667 | 0.5762 | 0.5333 | 0.1737 | 0.2110 |
| **Logistic Regression** | 0.5806 | 0.6550 | 0.1250 | 0.2500 | 0.1600 | 0.0429 | 0.0447 |
| **Gradient Boosting Classifier** | 0.5708 | 0.6608 | 0.5333 | 0.4750 | 0.4869 | 0.1164 | 0.1299 |
| **Quadratic Discriminant Analysis** | 0.5694 | 0.5417 | 0.4083 | 0.4850 | 0.3983 | 0.0781 | 0.1084 |
| **SVM - Linear Kernel** | 0.5444 | 0.0000 | 0.4750 | 0.2348 | 0.3094 | 0.0408 | 0.0551 |
| **Decision Tree Classifier** | 0.5431 | 0.5442 | 0.5833 | 0.4467 | 0.4970 | 0.0826 | 0.0766 |

Table S3 Classification Performance using mPerAF dataset

| **Model** | **Accuracy** | **AUC** | **Recall** | **Prec.** | **F1** | **Kappa** | **MCC** |
| --- | --- | --- | --- | --- | --- | --- | --- |
| **Logistic Regression** | 0.6875 | 0.6467 | 0.4000 | 0.5167 | 0.4210 | 0.2648 | 0.2815 |
| **CatBoost Classifier** | 0.6778 | 0.6600 | 0.3000 | 0.4250 | 0.3386 | 0.2117 | 0.2147 |
| **Extra Trees Classifier** | 0.6764 | 0.7333 | 0.2667 | 0.3167 | 0.2867 | 0.1875 | 0.1786 |
| **Ridge Classifier** | 0.6750 | 0.0000 | 0.6000 | 0.5333 | 0.5321 | 0.3061 | 0.3346 |
| **Decision Tree Classifier** | 0.6667 | 0.6467 | 0.5667 | 0.5950 | 0.5329 | 0.2850 | 0.3194 |
| **Extreme Gradient Boosting** | 0.6514 | 0.6333 | 0.4000 | 0.5400 | 0.4243 | 0.2077 | 0.2182 |
| **Random Forest Classifier** | 0.6292 | 0.5856 | 0.2000 | 0.3167 | 0.2367 | 0.0878 | 0.0956 |
| **Light Gradient Boosting Machine** | 0.6278 | 0.6200 | 0.4000 | 0.5733 | 0.4305 | 0.1723 | 0.1990 |
| **Gradient Boosting Classifier** | 0.6264 | 0.6156 | 0.4000 | 0.4333 | 0.3971 | 0.1585 | 0.1698 |
| **Naive Bayes** | 0.6250 | 0.6433 | 0.6333 | 0.4733 | 0.5325 | 0.2431 | 0.2539 |
| **Ada Boost Classifier** | 0.5889 | 0.5233 | 0.3333 | 0.3033 | 0.3033 | 0.0544 | 0.0558 |
| **K Neighbors Classifier** | 0.5806 | 0.5783 | 0.2667 | 0.3833 | 0.3033 | 0.0323 | 0.0302 |
| **SVM - Linear Kernel** | 0.5778 | 0.0000 | 0.4333 | 0.3150 | 0.3158 | 0.0935 | 0.1163 |
| **Linear Discriminant Analysis** | 0.5542 | 0.5789 | 0.5333 | 0.4267 | 0.4566 | 0.0937 | 0.1066 |
| **Quadratic Discriminant Analysis** | 0.4694 | 0.4367 | 0.3000 | 0.2633 | 0.2644 | -0.123 | -0.145 |

Table S4 Classification Performance using PerAF dataset

| **Model** | **Accuracy** | **AUC** | **Recall** | **Prec.** | **F1** | **Kappa** | **MCC** |
| --- | --- | --- | --- | --- | --- | --- | --- |
| **Linear Discriminant Analysis** | 0.7014 | 0.6833 | 0.6083 | 0.6317 | 0.6081 | 0.3702 | 0.3793 |
| **Extra Trees Classifier** | 0.6542 | 0.6500 | 0.2250 | 0.5000 | 0.3000 | 0.1578 | 0.1829 |
| **Ada Boost Classifier** | 0.6278 | 0.6200 | 0.4500 | 0.4833 | 0.4543 | 0.1843 | 0.1856 |
| **Logistic Regression** | 0.6181 | 0.5367 | 0.0000 | 0.0000 | 0.0000 | 0.0000 | 0.0000 |
| **Ridge Classifier** | 0.6181 | 0.0000 | 0.0000 | 0.0000 | 0.0000 | 0.0000 | 0.0000 |
| **K Neighbors Classifier** | 0.6153 | 0.5617 | 0.3083 | 0.4083 | 0.3483 | 0.1110 | 0.1085 |
| **Decision Tree Classifier** | 0.5778 | 0.5417 | 0.3833 | 0.4900 | 0.4038 | 0.0877 | 0.1061 |
| **Extreme Gradient Boosting** | 0.5778 | 0.5867 | 0.3417 | 0.4667 | 0.3802 | 0.0742 | 0.0775 |
| **Random Forest Classifier** | 0.5681 | 0.5942 | 0.1583 | 0.3333 | 0.2067 | -0.013 | 0.0071 |
| **Gradient Boosting Classifier** | 0.5681 | 0.6367 | 0.3833 | 0.4500 | 0.3943 | 0.0642 | 0.0755 |
| **Light Gradient Boosting Machine** | 0.5681 | 0.6000 | 0.2750 | 0.3833 | 0.3033 | 0.0201 | 0.0283 |
| **Naive Bayes** | 0.5458 | 0.5700 | 0.4833 | 0.4250 | 0.4169 | 0.0523 | 0.0749 |
| **CatBoost Classifier** | 0.5431 | 0.5650 | 0.1250 | 0.1750 | 0.1419 | -0.074 | -0.079 |
| **SVM - Linear Kernel** | 0.5181 | 0.0000 | 0.4000 | 0.1500 | 0.2182 | 0.0000 | 0.0000 |
| **Quadratic Discriminant Analysis** | 0.3958 | 0.3867 | 0.3333 | 0.1983 | 0.2452 | -0.227 | -0.252 |

Table S5 Classification Performance using Wavelet-ALFF dataset

| **Model** | **Accuracy** | **AUC** | **Recall** | **Prec.** | **F1** | **Kappa** | **MCC** |
| --- | --- | --- | --- | --- | --- | --- | --- |
| **K Neighbors Classifier** | 0.6444 | 0.6100 | 0.2833 | 0.5083 | 0.3376 | 0.1662 | 0.1937 |
| **Extreme Gradient Boosting** | 0.6333 | 0.6142 | 0.4833 | 0.5583 | 0.4833 | 0.2191 | 0.2379 |
| **Light Gradient Boosting Machine** | 0.6319 | 0.6537 | 0.4833 | 0.5933 | 0.4879 | 0.2196 | 0.2522 |
| **Ridge Classifier** | 0.6208 | 0.0000 | 0.3833 | 0.5917 | 0.4388 | 0.1716 | 0.1986 |
| **Linear Discriminant Analysis** | 0.6069 | 0.4817 | 0.4333 | 0.4967 | 0.4478 | 0.1526 | 0.1565 |
| **Naive Bayes** | 0.5833 | 0.5262 | 0.5417 | 0.5150 | 0.4938 | 0.1533 | 0.1693 |
| **CatBoost Classifier** | 0.5819 | 0.6150 | 0.1917 | 0.2333 | 0.2086 | 0.0268 | 0.0109 |
| **Logistic Regression** | 0.5722 | 0.5446 | 0.2667 | 0.3667 | 0.2938 | 0.0453 | 0.0442 |
| **Ada Boost Classifier** | 0.5458 | 0.6025 | 0.4583 | 0.3845 | 0.3955 | 0.0686 | 0.0962 |
| **Gradient Boosting Classifier** | 0.5347 | 0.6071 | 0.3000 | 0.3233 | 0.2800 | -0.008 | 0.0015 |
| **Random Forest Classifier** | 0.5222 | 0.5246 | 0.1667 | 0.2000 | 0.1800 | -0.082 | -0.097 |
| **Extra Trees Classifier** | 0.5222 | 0.5700 | 0.1917 | 0.2700 | 0.2122 | -0.067 | -0.070 |
| **SVM - Linear Kernel** | 0.5194 | 0.0000 | 0.4333 | 0.2028 | 0.2706 | -0.030 | -0.036 |
| **Decision Tree Classifier** | 0.5042 | 0.4942 | 0.4083 | 0.3469 | 0.3629 | -0.005 | -0.025 |
| **Quadratic Discriminant Analysis** | 0.4556 | 0.4358 | 0.3667 | 0.2633 | 0.3028 | -0.131 | -0.139 |
